# Perinatal Plasma Carotenoids and Vitamin E Concentrations with Glycemia and Insulin Resistance in Women during and after Pregnancy

**DOI:** 10.3390/nu15204421

**Published:** 2023-10-18

**Authors:** Jun S. Lai, Keith M. Godfrey, Choon Nam Ong, Kok Hian Tan, Fabian Yap, Yap Seng Chong, Jerry K. Y. Chan, Shiao-Yng Chan, Mary F.-F. Chong

**Affiliations:** 1 Singapore Institute for Clinical Sciences, Agency for Science Technology and Research, Singapore 117609, Singapore; obgcys@nus.edu.sg (Y.S.C.); obgchan@nus.edu.sg (S.-Y.C.); mary_chong@nus.edu.sg (M.F.-F.C.); 2 MRC Lifecourse Epidemiology Centre & NIHR Southampton Biomedical Research Centre, University of Southampton & University Hospital Southampton NHS Foundation Trust, Southampton SO16 6YD, UK; 3 Saw Swee Hock School of Public Health, National University of Singapore and National University Health System, Singapore 117549, Singapore; ephocn@nus.edu.sg; 4 Department of Maternal Fetal Medicine, KK Women’s and Children’s Hospital, Singapore 229899, Singapore; tan.kok.hian@singhealth.com.sg; 5 Department of Paediatric Endocrinology, KK Women’s and Children’s Hospital, Singapore 229899, Singapore; fabian.yap.k.p@singhealth.com.sg; 6 Duke-NUS Medical School, Singapore 169857, Singapore; 7 Department of Obstetrics & Gynaecology and Human Potential Translational Research Programme, Yong Loo Lin School of Medicine, National University of Singapore and National University Health System, Singapore 119228, Singapore; 8 Department of Reproductive Medicine, KK Women’s and Children’s Hospital, Singapore 229899, Singapore; jerrychan@duke-nus.edu.sg

**Keywords:** carotenoids, vitamin E, glycemia, insulin resistance, pregnancy, post-pregnancy

## Abstract

We examined the associations of perinatal plasma carotenoids and E vitamers concentrations with glycemia, insulin resistance, and gestational and type 2 diabetes mellitus during pregnancy and post-pregnancy in GUSTO women. Plasma carotenoid and E vitamer concentrations were measured at delivery, and principal component analysis was used to derive the patterns of their concentrations. Fasting and 2 h glucose levels and fasting insulin were measured at 26–28 weeks gestation and 4–6 years post-pregnancy, with the derivation of homeostatic model assessment for insulin resistance (HOMA-IR). In 678 women, two carotenoid patterns (CP1: α- and β-carotene and lutein; CP2: zeaxanthin, lycopene, and β-cryptoxanthin) and one E vitamer pattern (VE: γ-, δ-, and α-tocopherols) were derived. A higher CP1 score (1-SD) was associated with lower gestational fasting glucose (β (95%CI): −0.06 (−0.10, −0.02) mmol/L) and lower gestational (−0.17 (−0.82, 0.01) mmol/L, *p* = 0.06) and post-pregnancy HOMA-IR (−0.11 (−0.15, −0.08) mmol/L). A higher VE score (1 SD) was associated with higher gestational and post-pregnancy fasting and 2 h glucose (gestational: 0.05 (0.01, 0.08) and 0.08 (0.01, 0.16); post-pregnancy: 0.19 (0.07, 0.31) and 0.24 (0.06, 0.42) mmol/L). Higher α- and β-carotene and lutein may be beneficial for gestational fasting glycemia, but higher vitamin E may increase gestational and post-pregnancy glycemia, although these findings require confirmation in cohorts with prospective longitudinal measurements of these vitamins.

## 1. Introduction

During pregnancy, a high amount of circulating reactive oxygen species is generated by the placenta for optimal maternal adaptation to pregnancy and the normal development of the fetus [1]. These oxidative processes are counterbalanced by antioxidants to protect against oxidative damage. However, an imbalance between these oxidative processes and antioxidant capacity can lead to oxidative stress, which has adverse effects on pregnancy and fetal development [1]. Emerging evidence suggests that increased oxidative stress may be involved in the pathogenesis of gestational diabetes mellitus (GDM) [2].

Women who experience GDM are at higher risk of developing insulin resistance and T2DM later in life [3]; an estimated 15–25% of women with prior GDM develop T2DM 1–2 years after pregnancy, and 35–70% develop T2DM 10–15 years after pregnancy [4,5]. Asian populations are at a disproportionately higher risk of T2DM [6], and the prevalence of GDM (23.5%) in Singapore is among the highest in the world [7]. As such, potential interventions to prevent GDM and its progression to T2DM, including improving diet during the antenatal period, might have utility in reducing the lifetime risk of T2DM [8]. 

Dietary antioxidants such as carotenoids and vitamin E (comprising tocopherol and tocotrienol vitamers) are known to reduce oxidative stress [9,10]. Carotenoids can reduce reactive oxygen species, such as singlet oxygen and peroxyl radicals, as well as transcription factors, such as nuclear factor κB and nuclear factor erythroid 2-related factor 2 [9], which are responsible for insulin resistance and β-cell dysfunction [11]. Similarly, vitamin E especially, α-tocopherol, has been shown to reduce lipid peroxidation [10]; this produces superoxide, which damages the structural and functional components of the β cells crucial for maintaining normal glucose concentrations [11]. 

However, evidence relating carotenoids and vitamin E to glycemia and insulin resistance in pregnant women or to GDM is scarce. Two case–control studies have reported no differences in dietary β-carotene and vitamin E intake and no difference in serum α-tocopherol concentrations between women with and without GDM [12,13]. To the best of our knowledge, no studies have examined other carotenoids, such as lutein and zeaxanthin, or E vitamers in relation to glycemia and insulin resistance during pregnancy or investigated whether gestational plasma concentrations are related to women’s glycemia and insulin resistance post-pregnancy. 

There is increasing recognition that nutrients and dietary compounds have synergistic effects on health [14]. Thus, assessing combinations of dietary compounds using pattern analysis may be more appropriate for assessing the influence of highly correlated dietary compounds on glycemia. We aimed to examine the associations of individual plasma carotenoid and E vitamer concentrations at delivery and their combination with gestational glycemia, insulin resistance, and GDM, as well as glycemia, insulin resistance, and risk of T2DM 4–6 years post-pregnancy.

## 2. Materials and Methods

### 2.1. Study Sample

We analyzed data from the Growing Up in Singapore Towards healthy Outcomes (GUSTO) study, which is a prospective mother–offspring cohort in Singapore [15]. A detailed description of the study has been published [15]. In brief, pregnant women (≥18 years) of Chinese, Malay, or Indian ethnicity with homogenous parental ethnic background were recruited during their first trimester (<14 weeks) in June 2009–September 2010 from two major maternity hospitals (National University Hospital and KK Women’s and Children’s Hospital) in Singapore. All procedures of GUSTO were conducted according to the guidelines of the Declaration of Helsinki and received ethics approval from the institutional review board governing the two maternity hospitals. Written informed consent was obtained from all participants during recruitment. 

**Figure 1:**
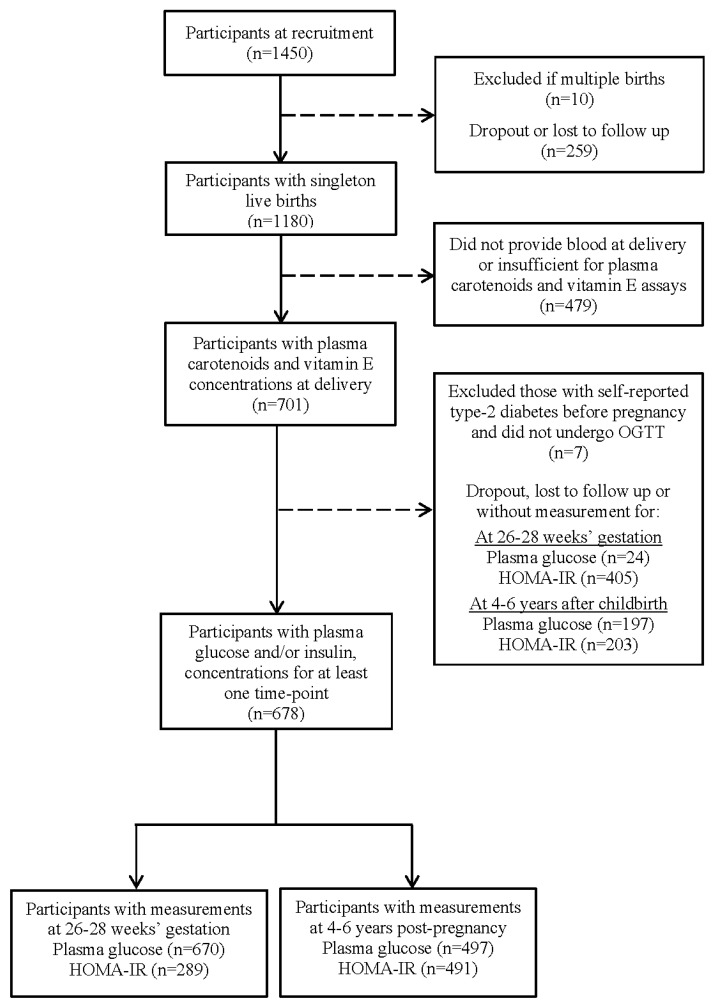
Participants included in the analysis of plasma carotenoid and vitamin E concentrations with glycemia and insulin during and after pregnancy in the Growing Up in Singapore Towards healthy Outcomes cohort. HOMA-IR, homeostatic model assessment for insulin resistance; OGTT, oral glucose tolerance test.

A total of 1450 pregnant women were recruited at baseline, and 1098 had singleton live births. The present analysis included all GUSTO women who provided sufficient blood for plasma carotenoid and E vitamer assays at delivery and had information on plasma glucose and/or insulin during pregnancy as well as plasma glucose and/or insulin 4–6 years post-pregnancy (Figure 1).

### 2.2. Plasma Concentrations of Carotenoids and E Vitamers

Non-fasting blood samples were collected from pregnant women (median gestation: 39 weeks, interquartile range: 38–40 weeks) around the time of delivery (up to 2 weeks prior or within 17 h after) by standard venipuncture. The blood samples were collected in EDTA tubes, centrifuged at 1600× *g* for 10 min at 4 °C within 4 h to obtain plasma, stored at −80 °C, and thawed prior to analysis. Ultra-high-performance liquid chromatography with photodiode array detection was used to determine plasma concentrations of carotenoids (α-carotene, β-carotene, β-cryptoxanthin, lutein, and zeaxanthin) and E vitamers (α-, γ-, and δ-tocopherols and tocotrienols) [16]. Method precision was examined using pooled and spiked plasma samples, and the results were similar to those previously published [16], with the relative standard deviations of within-day assays and between-day assays generally <10% and <15%, respectively. The half-lives of circulating carotenoids and E vitamers are 5–45 days [17,18,19] and 2–70 days [20], respectively; thus, maternal concentrations around the time of delivery reflect the concentrations in the last weeks of gestation.

### 2.3. Plasma Glucose and Insulin Concentrations, GDM, and T2DM

At 26–28 weeks gestation, we measured plasma glucose after an overnight fast and 2 h after a 75 g load in an oral glucose tolerance test (OGTT). Women with self-reported pre-existing T2DM before pregnancy were excluded from the OGTT. Fasting plasma insulin concentrations were measured in a subset of women with available fasting blood samples. Similarly, at 4–6 years post-pregnancy, fasting glucose, plasma glucose 2 h after an OGTT, and fasting plasma insulin were measured. Insulin and glucose concentrations were quantified using the colorimetry method (Advia 2400 Chemistry system, Siemens Medical Solutions Diagnostics; and Beckman LX20 Pro analyzer, Beckman Coulter, Brea, CA, USA). The HOMA-IR was calculated as (fasting plasma insulin × fasting plasma glucose)/22.5 [21].

GDM was defined as a plasma glucose concentration of ≥7.0 mmol/L fasting and/or ≥7.8 mmol/L 2 h post-OGTT, following the 1999 World Health Organization (WHO) standard criteria [22] in use for clinical management at that time. T2DM 4–6 years post-pregnancy was defined as a plasma glucose concentration ≥7.0 mmol/L fasting and/or ≥11.1 mmol/L 2 h post-OGTT, following the 2019 WHO classification of diabetes [23], or self-reported diagnosis of T2DM after GUSTO index pregnancy (*n* = 1).

### 2.4. Covariates

Covariates were selected according to the previous literature [12,13,24,25] and a directed acyclic graph. Information on the women’s age, ethnicity, highest education attained, self-reported existing T2DM, and family history of T2DM were collected during recruitment. The women’s pre-pregnancy body mass index (BMI) was calculated as weight divided by height squared (kg/m^2^) on the basis of self-reported pre-pregnancy weight and height measured with a stadiometer (SECA model 213) at 26–28 weeks gestation. Parity was retrieved from hospital delivery records. At 26–28 weeks gestation, self-reported cigarette smoking and alcohol intake during pregnancy were ascertained; moderate and vigorous physical activity in the past 7 days were self-reported using the International Physical Activity Questionnaire [26] and categorized as follows: never, <150, and ≥150 min/week. Food and dietary supplement intakes were assessed using a single 24 h recall administered by trained research staff. Total fat intake was estimated using nutrient analysis software (Dietplan 6, Forestfield Software, Horsham, UK) on the basis of a food composition database containing local foods [27]. The use of dietary supplements (yes/no) containing any amount of preformed vitamin A (retinol or retinyl esters), carotenoids, and vitamin E or its vitamers were considered.

### 2.5. Statistical Analysis

Descriptive statistics are presented for demographic, nutritional, and clinical measures for those included in the present analysis. 

To examine carotenoids and E vitamers in combination, we constructed patterns from six carotenoids and three E vitamers using principal component analysis with the use of varimax rotation. As a high percentage of participants had concentrations below the detection limit for each form of tocotrienol, all forms of tocotrienols were removed from subsequent analyses. The number of patterns chosen for retention was determined by the break point of the Scree plot and an eigenvalue of >1.0 (determined a priori). Differences in intake of fruit, vegetables, total fat, and dietary supplements between tertiles of carotenoid and E vitamer patterns were compared using chi-squared or one-way ANOVA tests.

To enable the comparison of effect estimates across exposures, we constructed standard deviation scores ((observed value − mean)/SD) for each carotenoid and E vitamer as well as the scores for their patterns. We assessed the associations of individual carotenoids and E vitamers and their patterns with (1) continuous measures of plasma glucose and the HOMA-IR (examined using linear regression for normal distributions and inverse Gaussian regressions for positively skewed distributions) and (2) categorical outcomes—GDM and T2DM (examined using logistic regression). All models were adjusted for the women’s age at delivery, ethnicity, education, pre-pregnancy overweight or obesity (BMI ≥ 23 kg/m^2^ according to WHO BMI classification for Asian [28]), family history of diabetes mellitus, parity, smoking, alcohol intake, moderate–strenuous physical activity, total fat intake, and dietary supplement intake. 

Missing data for covariates were imputed using multiple imputation with chained equations (20 times) for the following confounding variables: highest education attained (*n* = 5), pre-pregnancy BMI (*n* = 56), family history of T2DM (*n* = 10), smoking (*n* = 5), alcohol intake (*n* = 19), physical activity (*n* = 4), total fat intake (*n* = 10), and dietary supplement intake (*n* = 47). All analyses were performed using Stata version 14 (StataCorp LP, College Station, TX, USA). Two-sided *p* < 0.05 was considered statistically significant.

## 3. Results

### 3.1. Study Sample Characteristics

Table 1 presents the demographic, lifestyle, and clinical characteristics along with the average concentrations of individual carotenoids and E vitamers for the 678 women included. The women were, on average, 31 years old at delivery. Most were of Chinese ethnicity (59%), had attained tertiary education (37.4%), were multiparous (56.2%) at recruitment, and did not engage in moderate–vigorous physical activity (70%). Approximately 39.6% of women had overweight or obesity before pregnancy, 2% smoked and drank alcohol during pregnancy, and 73.5% and 26.8% of women took dietary supplements containing vitamin A/carotenoids and vitamin E in mid–late pregnancy, respectively. A total of 204 (30.5%) women reported a family history of T2DM, 130 (19.4%) were classified as having GDM, and 11 (2.2%) were classified as having T2DM 4–6 years post-pregnancy.

### 3.2. Patterns of Carotenoid and E Vitamers

Three patterns were derived (Table 2). Carotenoid pattern 1 (CP1) was represented by α-carotene, β-carotene, and lutein; the vitamin E (VE) pattern consisted of all forms of tocopherols (γ-, δ-, and α-tocopherols); and carotenoid pattern 2 (CP2) comprised zeaxanthin, lycopene, and β-cryptoxanthin.

We found that women who were in the highest tertile of CP1 scores had significantly higher fruit and vegetable intake (Table 3). Only total fat intake was significantly different according to the tertiles of VE scores, whilst only vegetable intake was significantly different according to the tertiles of CP2 scores (higher intake for those in highest tertiles). There were no significant differences in the proportion of women consuming dietary supplements containing vitamin A and carotenoids or containing vitamin E according to the patterns scores. 

### 3.3. Carotenoid and E Vitamers with Gestational Plasma Glucose, HOMA-IR, and GDM

Higher α- and β-carotene and lutein concentrations (per SD increment), examined individually, were significantly associated with 0.05 mmol/L (95% CI: −0.09, −0.01), 0.06 mmol/L (95% CI: −0.08, −0.01), and 0.05 mmol/L (95% CI: −0.09, −0.01) decreases in gestational fasting glucose, respectively (Table 4). Likewise, the combination of α- and β-carotene and lutein was inversely associated with gestational fasting glucose, as reflected by a higher CP1 score (per SD increment) significantly associated with a 0.06 mmol/L (95% CI: −0.10, −0.02; *p* = 0.004) lower gestational fasting glucose. There was a trend toward an association between a higher CP1 score (combination of α- and β-carotene and lutein) and a lower gestational HOMA-IR (β −0.17, 95% CI: −0.82, 0.01), but the association was borderline significant (*p* = 0.06) due to the small sample size. Individual concentrations of α- and β-carotene and lutein were not significantly associated with gestational HOMA-IR. No statistically significant associations were observed for individual carotenoids and their combinations with gestational 2 h glucose and the likelihood of GDM.

Additionally, there was a trend toward an association between higher β-cryptoxanthin concentrations and a lower gestational HOMA-IR (β −0.09, 95% CI: −0.72, 0.01 per SD increment in concentrations), but the association was borderline significant (*p* = 0.06) due to the small sample size. The combination of zeaxanthin, lycopene, and β-cryptoxanthin was not associated with gestational HOMA-IR. Overall, zeaxanthin, lycopene, and β-cryptoxanthin, whether examined individually or in combination (CP2), were not associated with gestational fasting or 2 h glucose or the likelihood of GDM. 

Individually, higher γ-tocopherol concentrations (per SD increment) were associated with higher gestational glucose concentrations (fasting 0.05 mmol/L (95% CI: 0.02, 0.09), 2 h 0.10 mmol/L (95% CI: 0.02, 0.17)). Additionally, higher δ-tocopherol (per SD increment) was associated with a 0.05 mmol/L (95% CI: 0.02, 0.09) increase in gestational fasting glucose but was not significantly associated with 2 h glucose. The combination of γ-, δ-, and α-tocopherols (VE pattern) was associated with higher gestational glucose concentrations (fasting 0.05 mmol/L (95% CI: 0.01, 0.08), 2 h 0.08 mmol/L (95% CI: 0.01, 0.16) per SD score increment).

No statistically significant association was observed for α-tocopherol with gestational fasting or 2 h glucose. Individual E vitamers and their combinations were not associated with the HOMA-IR or the likelihood of GDM.

### 3.4. Carotenoid and E Vitamers with Post-Pregnancy Plasma Glucose, HOMA-IR, and T2DM

Higher β-carotene concentrations (per SD increment) were associated with a 0.05 (95% CI: −0.07, −0.04) decrease in the HOMA-IR score (Table 5). A higher score in the combination of α- and β-carotene and lutein (per SD increment in CP1 score), was also associated with a 0.11 (95% CI: −0.15, −0.08) decrease in the HOMA-IR score. No statistically significant associations were observed for individual α-carotene or lutein with the HOMA-IR.

Additionally, higher β-cryptoxanthin concentrations (per SD increment) were associated with a 0.07 (95% CI: −0.13, −0.02) decrease in the HOMA-IR score. However, neither individual zeaxanthin and lycopene nor the combination of zeaxanthin, lycopene, and β-cryptoxanthin (CP2) were not associated with the HOMA-IR.

Individual carotenoids and their combinations were not associated with post-pregnancy fasting or 2 h glucose, nor with the risk of T2DM. 

Individually, higher δ-tocopherol concentrations (per SD increment) were associated with higher glucose concentrations (fasting 0.15 mmol/L (95% CI: 0.03, 0.27) and 2 h 0.26 mmol/L (95% CI: 0.08, 0.44)). Additionally, higher γ-tocopherol concentrations (per SD increment) were associated with a 0.21 mmol/L (95% CI: 0.09, 0.33) increase in fasting glucose but were not significantly associated with 2 h glucose. The combination of γ-, δ-, and α-tocopherols (VE pattern) was associated with higher glucose concentrations ((fasting 0.19 mmol/L (95% CI: 0.07, 0.31) and 2 h 0.24 mmol/L (95% CI: 0.06, 0.42) per SD increment in VE pattern score). 

No statistically significant associations were observed for α-tocopherol with post-pregnancy glucose, and there were no associations of E vitamers and their pattern with the HOMA-IR or T2DM.

## 4. Discussion

This study found associations of higher late-pregnancy concentrations of α- and β-carotene and lutein (in combination) with lower fasting glucose during pregnancy, as well as a lower HOMA-IR during pregnancy and 4–6 years post-pregnancy. Additionally, a higher late-pregnancy β-cryptoxanthin concentration was individually associated with a lower HOMA-IR during pregnancy and 4–6 years post-pregnancy. By contrast, higher concentrations of γ-, δ-, and α-tocopherols in combination were associated with higher fasting and 2 h glucose during pregnancy and post-pregnancy but not with the HOMA-IR.

The associations we observed for α- and β-carotene (when examined individually) with plasma glucose during pregnancy are reminiscent of those in studies on non-pregnant populations. Overall, the effect size of the lowered fasting glucose during pregnancy we found for a 1 SD increment in α- or β-carotene concentrations (0.05–0.06 mmol/L) was approximately the same as that found in studies on non-pregnant populations (0.05–0.25 mmol/L) [29,30]. However, our finding that a higher lutein concentration was associated with lower fasting glucose but not 2 h glucose during pregnancy is in direct contrast to a study in a non-pregnant population, which observed significant associations with lower 2 h glucose but not fasting glucose [29]. When α- and β-carotene and lutein were examined in combination (CP1), the association with fasting glucose during pregnancy was also significant.

We additionally found that higher β-cryptoxanthin concentrations were associated with a lower HOMA-IR during pregnancy (although this was a trending association due to the small sample size) and 4–6 years post-pregnancy, which contrasted studies showing no significant association between serum β-cryptoxanthin concentrations and the HOMA-IR in non-pregnant populations [24,29]. The difference in findings may be due to variations in carotenoid concentrations influenced by the pregnancy-related hyperlipidemic state, placental transfer, or consumption of foods rich in β-cryptoxanthin (e.g., a greater consumption of tropical fruits in Singapore [31], which are rich sources of β-cryptoxanthin [32]).

The magnitude of the above associations may appear modest (0.06 mmol/L lower fasting glucose), but such an effect size was associated with an appreciable reduction in the odds of delivery by cesarean section, delivery of a neonate with a birth weight > 90th percentile, neonatal hypoglycemia, and fetal hyperinsulinemia in the Hyperglycemia and Adverse Pregnancy Outcome (HAPO) study [33], and it is thus considered clinically impactful.

Our findings support the beneficial role of combined α- and β-carotene and lutein (CP1) in insulin resistance (HOMA-IR) during pregnancy (albeit borderline significance due to the small sample size) and post-pregnancy. The significant association with the post-pregnancy HOMA-IR could be mediated through a lowered gestational HOMA-IR, changes to the physiological function of tissues influencing insulin resistance that persist beyond pregnancy, or consistent adherence to a diet high in these carotenoids post-pregnancy. Further studies specifically designed to address this will be needed. Although we observed associations with the HOMA-IR at both time points, the beneficial associations of these carotenoids with gestational fasting glucose did not persist beyond pregnancy. One possible explanation could be a heightened state of physiological insulin resistance during pregnancy compared with a milder state of insulin resistance post-pregnancy. The heightened state of insulin resistance during pregnancy returns to the pre-pregnancy state after delivery, and as such, these women may not be sufficiently resistant to insulin 4–6 years post-pregnancy to observe an appreciable change in glycemia. Our observation of consistent associations between CP1 and HOMA-IR during pregnancy as well as post-pregnancy but less consistent associations when these carotenoids were examined individually further supports the value of examining combinations of carotenoids to account for their synergistic activities.

We observed differences in fruit and vegetable intakes according to tertiles of CP1 scores but no significant differences in the proportion of individuals consuming vitamin A/carotenoid supplements. Additionally, in the same cohort, we previously found women with higher concentrations of β-cryptoxanthin to have a higher intake of fruit and vegetables during pregnancy [34], but the concentrations did not differ significantly by intake of vitamin A/carotenoid supplements [35]. These women were also found to have lower mean concentrations of α-carotene, β-cryptoxanthin, and lutein compared with other pregnant cohorts, even with approximately 70% of them consuming vitamin A/carotenoid-containing supplements, possibly because of lower fruit and vegetable intake in the cohort or because the dietary supplements consumed did not contain the full range of carotenoids [35]. Taken together, our findings suggest that greater consumption of fruit and vegetables but not dietary supplements containing vitamin A/carotenoids is beneficial for lowering glycemia and insulin resistance during and/or after pregnancy. Fruit and vegetables, apart from their antioxidative properties, are also higher in fiber and lower in glycemic index, which aligns with existing dietary approaches for glycemic control in non-pregnant populations [36]. Continued health promotion efforts encouraging higher fruit and vegetable consumption in pregnant women are needed for better glycemic outcomes during and after pregnancy.

Our analysis may be underpowered to detect statistically significant differences in 2 h glucose concentrations (due to a much wider variation in concentrations compared with fasting glucose), as well as in the odds of GDM and T2DM (categorical variables with a small number of cases). Further investigations in studies with larger sample sizes are needed.

In contrast to carotenoids, γ-, δ-, and α-tocopherols in combination were associated with higher fasting and 2 h glucose during pregnancy. This association is likely driven by γ- and δ-tocopherols, considering their higher loadings in this pattern as well as their individual associations with plasma glucose. However, the literature is sparse on the mechanism of action linking γ- and δ-tocopherols to glucose metabolism. There is some evidence suggesting that γ-tocopherol may be pro-inflammatory [37]; as such, the positive associations with plasma glucose and GDM may be a result of increased inflammation—one of the underlying mechanisms of hyperglycemia during pregnancy [38]. Alternatively, higher concentrations of γ- and δ-tocopherols may reflect the widespread use of refined blended plant oils around the world, which have a very high content of γ-tocopherol [39]. One study in Chinese adults found higher intakes of refined blended plant oils to be associated with an increased risk of T2DM, likely because frying is the predominant cooking method adopted for this type of oil [40] or because of its higher content of *n*-6 fatty acids, which play conflicting roles in glucose metabolism [41]. While our study also observed that those who were in the highest tertile of CP2 scores had significantly higher total fat intake, a thorough evaluation of γ- or δ-tocopherol concentrations and their food sources as well as a comprehensive understanding of their individual cellular actions and impact on oxidative stress are needed to elucidate the impact of the different E vitamers on glucose metabolism.

The strengths of this study are that it is the first to relate maternal plasma concentrations of individual carotenoids and E vitamers to glycemic measures during pregnancy and a few years post-pregnancy and that it derived carotenoid and E vitamer combinations to capture their synergistic activities. Several limitations are noted. The blood samples (around the time of delivery) used for plasma carotenoid and vitamin E assays were taken after the measurement of plasma glucose during pregnancy (26–28 weeks gestation), and as such, reverse causation cannot be ruled out. However, studies have shown stability in carotenoid and α- and γ-tocopherol concentrations from the second trimester to delivery [42,43] as well as stability in dietary patterns throughout pregnancy [44,45]. The use of non-fasting plasma samples may have introduced systematic bias, but studies have shown non-significant differences in carotenoid concentrations pre- and post-meal [46,47]; the effect of fasting compared with non-fasting is less clear for plasma E vitamers due to limited literature. Findings regarding the gestational HOMA-IR were also limited by the smaller sample size, as more than half of the cohort did not provide sufficient fasting blood samples for insulin measurements. While the aim of the study was to examine the influences of carotenoids and vitamin E during the perinatal period, our study could have benefited from having measurements of carotenoid and vitamin E concentrations 4–6 years post-pregnancy.

## 5. Conclusions

Our study showed that higher maternal concentrations of α- and β-carotene and lutein are associated with a lower fasting glucose and HOMA-IR during pregnancy as well as a lower HOMA-IR 4–6 years post-pregnancy. These findings allude to the importance of consuming a greater quantity and variety of fruits and vegetables during pregnancy to ensure sufficient concentrations of a range of carotenoids (α- and β-carotene, lutein, and β-cryptoxanthin); however, these findings will require confirmation in other similar cohorts with prospective, longitudinal measurements of carotenoids and E vitamers, plasma glucose, and insulin during and after pregnancy. The replication of these findings in populations of different ethnic characteristics and with different dietary patterns is also required before recommendations can be made. Our finding that higher concentrations of γ- and δ-tocopherols were associated with higher plasma glucose during pregnancy highlights the need to further investigate specific E vitamers and their roles in metabolic health.

## Figures and Tables

**Table 1:** Characteristics ^a^ of participants for the associations of plasma carotenoids and vitamin E concentrations with glycemia and insulin resistance during and after pregnancy in the Growing Up in Singapore Towards healthy Outcomes cohort (*n* = 678).

Characteristics	*n* (%)
Pre-pregnancy overweight/obese (BMI ≥ 23.0 kg/m^2^), *n* (%)	246 (39.6)
During pregnancy	
Age at delivery, year, mean ± SD	31.4 ± 5.0
Ethnicity, *n* (%)	
Chinese	400 (59.0)
Malay	158 (23.3)
Indian	120 (17.7)
Highest education, *n* (%)	
≤Secondary	203 (30.2)
Post-secondary	218 (32.4)
Tertiary	252 (37.4)
Parity, *n* (%)	
Nulliparous	297 (43.8)
Primi-/Multiparous	381 (56.2)
Smoking, *n* (%)	13 (1.9)
Alcohol intake, *n* (%)	15 (2.3)
Moderate–vigorous physical activity, *n* (%)	
Never	472 (70.0)
<150 min/week	135 (20.0)
≥150 min/week	67 (9.9)
Total fat intake, g/day, mean ± SD	69.6 ± 29.1
Intake of supplements, *n* (%), containing	
Vitamin A/carotenoids	462 (73.5)
Vitamin E	169 (26.8)
Plasma carotenoids concentrations, μmol/L, mean ± SD	
α-Carotene	0.12 ± 0.09
β-Carotene	0.45 ± 0.36
β-Cryptoxanthin	0.45 ± 0.33
Lutein	0.46 ± 0.26
Zeaxanthin	0.30 ± 0.12
Lycopene	0.23 ± 0.13
Plasma vitamin E concentrations, μmol/L, mean ± SD	
α-tocopherol	52.45 ± 13.09
γ-tocopherol	1.47 ± 0.77
δ-tocopherol	0.47 ± 0.29
Family history of diabetes mellitus, *n* (%)	204 (30.5)
Fasting plasma glucose, mmol/L, mean ± SD	4.35 ± 0.49
2 h plasma glucose, mmol/L, mean ± SD	6.56 ± 1.54
HOMA-IR, median (IQR)	1.18 (0.80, 1.70)
Gestational diabetes, *n* (%)	130 (19.4 ^b^)
At 4–6 years post-pregnancy	
Fasting plasma glucose, mmol/L, mean ± SD	4.94 ± 0.73
2 h plasma glucose, mmol/L, mean ± SD	6.36 ± 1.89
HOMA-IR, median (IQR)	1.22 (0.81, 1.97)
Type 2 diabetes, *n* (%)	11 (2.2 ^c^)

^a^ Characteristics were based on data obtained during pregnancy or 4–6 years post-pregnancy unless otherwise specified. Missing data: *n* = 5 highest education attained, *n* = 56 pre-pregnancy BMI, *n* = 10 family history of T2DM, *n* = 5 smoking, *n* = 19 alcohol, *n* = 4 physical activity, *n* = 10 total fat intake, and *n* = 47 dietary supplements intakes. ^b^ Percentage calculated based on 670 women with plasma glucose concentrations at 26–28 weeks gestation. ^c^ Percentage calculated based on 497 women with plasma glucose concentrations 4–6 years post-pregnancy.

**Table 2:** Carotenoid and E vitamer pattern construction: Pattern structure and variance explained ^a^.

Carotenoid/E Vitamers	Carotenoid Pattern 1 (CP1)	Vitamin E (VE) Pattern	Carotenoid Pattern 2 (CP2)
α-carotene	0.56		
β-carotene	0.51		
lutein	0.48		
γ-tocopherol		0.61	
δ-tocopherol		0.60	
α-tocopherol		0.42	
zeaxanthin			0.59
lycopene			0.55
β-cryptoxanthin			0.46
% variance explained by each pattern	21.9	20.5	17.1
Cumulative % of variance explained	21.9	42.4	39

Values are loading coefficients derived from principal component analysis (^a^ absolute values < 0.30 are not listed for simplicity).

**Table 3:** Intake of fruit, vegetables, total fat, and dietary supplements according to tertiles of carotenoid and E vitamer pattern scores.

	Carotenoid Pattern 1 (CP1)		*p*	Vitamin E (VE) Pattern		*p*	Carotenoid Pattern 2 (CP2)		*p*
	Tertile 1	Tertile 3		Tertile 1	Tertile 3		Tertile 1	Tertile 3	
Total fruit intake, g/day, mean ± SD	63.7 ± 119.4	163.1 ± 189.7	<0.001	122.1 ± 171.1	104.2 ± 165.4	0.091	99.0 ± 144.6	127.6 ± 185.9	0.379
Total vegetables intake, g/day, mean ± SD	56.0 ± 64.2	80.6 ± 74.9	0.001	66.7 ± 69.5	67.6 ± 70.6	0.095	59.0 ± 67.8	73.7 ± 69.7	0.029
Total fat intake, g/day, mean ± SD	67.3 ± 29.3	72.5 ± 28.2	0.157	68.3 ± 30.6	73.8 ± 28.9	0.020	68.1 ± 26.7	71.4 ± 30.5	0.574
Supplements containing Vitamin A, *n* (%)	167 (75.9)	146 (70.9)	0.499	164 (76.3)	150 (69.4)	0.233	161 (75.2)	156 (74.3)	0.599
Supplements containing Vitamin E, *n* (%)	49 (22.3)	65 (31.6)	0.097	63 (29.3)	47 (21.8)	0.107	61 (28.5)	52 (24.8)	0.669

*p*-Values were obtained from chi-squared or one-way ANOVA tests.

**Table 4:** Associations of individual carotenoids and E vitamers and their patterns ^a^ in late pregnancy with plasma glucose, HOMA-IR during pregnancy, and GDM in the Growing Up in Singapore Towards healthy Outcomes cohort ^b,c^.

	Fasting Glucose (*n* = 670)		2 h Glucose (*n* = 670)		HOMA-IR (*n* = 289)		GDM (*n* = 130) vs. non-GDM (*n* = 540)	
	β (95% CI)	*p*	β (95% CI)	*p*	β (95% CI)	*p*	OR (95% CI)	*p*
Carotenoids ^d^								
Individual concentrations								
α-Carotene	−0.05 (−0.09, −0.01)	**0.005**	−0.13 (−0.24, 0.01)	0.05	−0.05 (−0.41, 0.32)	0.18	0.89 (0.71, 1.12)	0.33
β-Carotene	−0.06 (−0.08, −0.01)	**0.018**	−0.03 (−0.15, 0.10)	0.68	−0.12 (−0.46, 0.01)	0.07	1.11 (0.90, 1.35)	0.32
Lutein	−0.05 (−0.09, −0.01)	**0.019**	0.02 (−0.11, 0.15)	0.76	−0.01 (−0.26, 0.24)	0.95	1.07 (0.85, 1.34)	0.56
CP 1	−0.06 (−0.10, −0.02)	**0.004**	−0.04 (−0.17, 0.08)	0.50	−0.17 (−0.82, 0.01)	0.06	1.05 (0.84, 1.33)	0.65
Individual concentrations								
Zeaxanthin	−0.02 (−0.06, 0.01)	0.22	−0.08 (−0.20, 0.03)	0.17	−0.08 (−0.35, 0.12)	0.10	0.94 (0.74, 1.18)	0.59
Lycopene	−0.02 (−0.05, 0.02)	0.42	−0.05 (−0.16, 0.06)	0.39	0.03 (−0.08, 0.15)	0.58	1.09 (0.89, 1.33)	0.41
β-Cryptoxanthin	−0.04 (−0.05, 0.01)	0.05	0.02 (−0.10, 0.13)	0.80	−0.09 (−0.72, 0.01)	0.06	1.01 (0.81, 1.26)	0.91
CP 2	−0.04 (−0.06, 0.01)	0.05	−0.07 (−0.19, 0.05)	0.23	−0.01 (−0.43, 0.20)	0.11	1.02 (0.82, 1.27)	0.86
Vitamin E ^e^								
Individual concentrations								
γ-Tocopherol	0.05 (0.02, 0.09)	**0.004**	0.10 (0.02, 0.17)	**0.010**	0.09 (−0.24, 0.43)	0.57	1.20 (1.00, 1.50)	0.06
δ-Tocopherol	0.05 (0.02, 0.09)	**0.006**	0.07 (−0.01, 0.15)	0.07	0.03 (−0.34, 0.23)	0.11	1.22 (0.99, 1.49)	0.06
α-Tocopherol	−0.001 (−0.04, 0.04)	0.94	0.02 (−0.06, 0.10)	0.55	−0.05 (−0.58, 0.02)	0.07	1.21 (0.99, 1.49)	0.07
VE pattern	0.05 (0.01, 0.08)	**0.015**	0.08 (0.01, 0.16)	**0.033**	0.03 (−0.26, 0.34)	0.80	1.19 (0.99, 1.58)	0.06

GDM, gestational diabetes mellitus; HOMA-IR, homeostatic model assessment for insulin resistance. ^a^ CP 1: α- and β-carotene and lutein; CP 2: zeaxanthin, lycopene, and β-cryptoxanthin; VE pattern: γ-, δ-, and α-tocopherols. ^b^ Effect estimates are per SD increment in pattern score or individual carotenoid and vitamin E concentrations (*p* < 0.05 in bold). ^c^ All models adjusted for age, ethnicity, education, pre-pregnancy overweight and obese status, parity at recruitment, family history of T2DM, and the following at mid–late pregnancy: smoking status, alcohol intake, moderate–strenuous physical activity, total fat intake, and intake of any supplements containing ^d^ vitamin A/carotenoids and/or ^e^ vitamin E.

**Table 5:** Associations of individual carotenoids and E vitamers and their patterns ^a^ in late pregnancy with plasma glucose and HOMA-IR as well as T2DM 4–6 years post-pregnancy in the Growing Up in Singapore Towards healthy Outcomes cohort ^b,c.^.

	Fasting Glucose (*n* = 497)		2 h Glucose (*n* = 497)		HOMA-IR (*n* = 491)		T2DM (*n* = 11) vs. non-T2DM (*n* = 486)	
	β (95% CI)	*p*	β (95% CI)	*p*	β (95% CI)	*p*	OR (95% CI)	*p*
Carotenoids ^d^								
Individual concentrations								
α-carotene	−0.02 (−0.09, 0.05)	0.60	−0.03 (−0.21, 0.16)	0.77	−0.06 (−0.13, 0.01)	0.08	0.29 (0.07, 1.20)	0.09
β-carotene	−0.03 (−0.10, 0.04)	0.40	−0.04 (−0.22, 0.14)	0.68	−0.05 (−0.07, −0.04)	**0.001**	0.41 (0.09, 1.78)	0.23
Lutein	−0.01 (−0.10, 0.07)	0.76	0.01 (−0.20, 0.21)	0.94	−0.05 (−0.11, 0.01)	0.11	0.53 (0.17, 1.65)	0.28
CP 1	−0.02 (−0.10, 0.06)	0.64	−0.08 (−0.27, 0.12)	0.42	−0.11 (−0.15, −0.08)	**0.001**	0.32 (0.10, 1.00)	0.05
Individual concentrations								
Zeaxanthin	−0.04 (−0.07, 0.01)	0.06	−0.17 (−0.33, 0.01)	0.06	−0.07 (−0.14, 0.01)	0.06	0.35 (0.19, 1.31)	0.06
Lycopene	−0.04 (−0.12, 0.06)	0.56	0.01 (−0.19, 0.21)	0.91	−0.07 (−0.15, 0.01)	0.10	0.81 (0.38, 1.74)	0.60
β-cryptoxanthin	−0.02 (−0.10, 0.05)	0.55	−0.10 (−0.28, 0.07)	0.25	−0.07 (−0.13, −0.02)	**0.009**	0.36 (0.10, 1.26)	0.11
CP 2	−0.04 (−0.08, 0.01)	0.06	−0.08 (−0.26, 0.11)	0.41	−0.09 (−0.16, 0.02)	0.10	0.42 (0.17, 1.02)	0.06
Vitamin E ^e^								
Individual concentrations								
γ-Tocopherol	0.21 (0.09, 0.33)	**0.001**	0.17 (−0.01, 0.34)	0.06	−0.02 (−0.11, 0.06)	0.62	1.16 (0.65, 2.10)	0.62
δ-Tocopherol	0.15 (0.03, 0.27)	**0.015**	0.26 (0.08, 0.44)	**0.006**	0.01 (−0.06, 0.15)	0.34	1.47 (0.85, 2.54)	0.17
α-Tocopherol	0.10 (−0.02, 0.22)	0.10	0.08 (−0.12, 0.27)	0.43	0.05 (−0.04, 0.15)	0.24	0.73 (0.35, 1.50)	0.39
VE pattern	0.19 (0.07, 0.31)	**0.002**	0.24 (0.06, 0.42)	**0.009**	0.10 (−0.03, 0.24)	0.13	1.25 (0.67, 2.34)	0.48

GDM, gestational diabetes mellitus; HOMA-IR, homeostatic model assessment for insulin resistance; T2DM, type-2 diabetes mellitus. ^a^ CP 1: α- and β-carotene and lutein; CP 2: zeaxanthin, lycopene, and β-cryptoxanthin; VE pattern: γ-, δ-, and α-tocopherols. ^b^ Effect estimates are per SD increment in pattern scores or individual carotenoids and vitamin E concentrations (*p* < 0.05 in bold). ^c^ All models adjusted for age, ethnicity, education, pre-pregnancy overweight and obese status, parity at recruitment, family history of T2DM, and the following at mid–late pregnancy: smoking status, alcohol intake, moderate–strenuous physical activity, total fat intake, and intake of any supplements containing ^d^ vitamin A/carotenoids and/or ^e^ vitamin E.

## Data Availability

The data described in the manuscript will be made available upon request pending approval by lead investigators of the GUSTO study.
